# Characterization of Effects of Compressed Sensing on High Spectral and Spatial Resolution (HiSS) MRI with Comparison to SENSE

**DOI:** 10.3390/tomography9020055

**Published:** 2023-03-19

**Authors:** Milica Medved, Marco Vicari, Gregory S. Karczmar

**Affiliations:** 1 Department of Radiology, University of Chicago, Chicago, IL 60637, USA; 2 Fraunhofer Institute for Digital Medicine MEVIS, 28359 Bremen, Germany; 3 Philips Research, 5656 AE Eindhoven, The Netherlands

**Keywords:** compressed sensing (CS), high spectral and spatial resolution (HiSS) MRI, image quality, modulation transfer function, spectral lineshape, dispersion vs. absorption (DISPA) analysis

## Abstract

High Spectral and Spatial resolution (HiSS) MRI shows high diagnostic performance in the breast. Acceleration methods based on k-space undersampling could allow stronger T2*-based image contrast and/or higher spectral resolution, potentially increasing diagnostic performance. An agar/oil phantom was prepared with water-fat boundaries perpendicular to the readout and phase encoding directions in a breast coil. HiSS MRI was acquired at 3T, at sensitivity encoding (SENSE) acceleration factors R of up to 10, and the R = 1 dataset was used to simulate corresponding compressed sensing (CS) accelerations. Image quality was evaluated by quantifying noise and artifact levels. Effective spatial resolution was determined via modulation transfer function analysis. Dispersion vs. absorption (DISPA) analysis and full width at half maximum (FWHM) quantified spectral lineshape changes. Noise levels remained constant with R for CS but amplified with SENSE. SENSE preserved the spatial resolution of HiSS MRI, while CS reduced it in the phase encoding direction. SENSE showed no effect on FWHM or DISPA markers, while CS increased FWHM. Thus, CS might perform better in noise-limited or geometrically constrained applications, but in geometric configurations specific to breast MRI, spectral analysis might be compromised, decreasing the diagnostic performance of HiSS MRI.

## 1. Introduction

High Spectral and Spatial resolution (HiSS) MRI is a high spatial resolution spectroscopic imaging sequence designed for applications in body imaging. Rather than quantifying the relative intensities of metabolites in water-suppressed spectra acquired in centimeter-sized voxels, as is a common current application of spectroscopic imaging, HiSS MRI can be used to examine the spectral structure of the water and lipid resonances in sub-millimeter-sized voxels [1,2]. While conventional imaging assumes that the water resonance is well represented with a single Lorentzian, corresponding to a single uniform water tissue compartment, HiSS MRI has demonstrated that the water resonance often shows inhomogeneous broadening, revealing multiple components, corresponding to multiple sub-voxel tissue compartments [3,4,5]. Information on these sub-voxel compartments, and their differences between healthy and diseased tissue, can be leveraged for diagnostic purposes [6,7,8,9,10].

HiSS MRI has primarily been applied to non-contrast breast imaging, with improved fat suppression, high image quality, and high lesion characterization performance [11,12,13,14,15]. This has been demonstrated when using both the morphological water peak height (WPH) images [11,16,17] and the analysis of the spectral characteristics of the lesion voxel signal [6,7,8,9]. Thus, HiSS MRI has shown the potential to serve as a powerful tool for non-contrast enhanced breast cancer MRI screening. The development of such an imaging protocol is an ongoing area of research and would open up MRI breast cancer screening to a wider population of women, potentially making it as ubiquitous as the current X-ray-based mammographic screening [18,19,20,21,22,23,24,25,26,27]. However, the spectroscopic nature of HiSS MRI results in relatively long acquisition times. In breast imaging, this has been mitigated with the use of SENSE acceleration [15,28,29]. Shorter echo trains have also been necessary, resulting in reduced image contrast and spectral resolution, which can affect image quality [30]. Compressed sensing (CS) is an alternative acceleration technique that is well-established in MR imaging [31,32]. CS algorithms specific to spectroscopic imaging have been used successfully for ^13^C and ^31^P nuclei, with applications to ^1^H spectroscopic imaging that is conducted on common clinical scanners being complicated by the lack of sparsity of the ^1^H spectrum, as well as the presence of strong water and lipid resonances obscuring the metabolites of interest [33]. In HiSS MRI, however, the water and fat spectral resonances are of interest. The standard CS algorithm is applied in spatial (not spectral) dimensions, so these concerns are not expected to reduce performance. In such conventional applications, CS can be expected to provide high acceleration factors with noise suppression and lower artifact levels. Still, the advantages of CS relative to SENSE acceleration methods for HiSS MRI are unclear.

Successful implementation of high k-space undersampling-based acceleration methods would allow for fewer k-space lines needed. The resulting acquisition time savings could be used as a trade-off to allow longer echo trains necessary for spectral/spatial imaging, producing stronger T2*-based image contrast and/or higher spectral resolution in HiSS MRI, potentially increasing sensitivity and diagnostic performance. Additionally, the implementation of CS would increase the utility of HiSS MRI in other applications where SENSE acceleration is constrained by coil geometry, such as in prostate imaging [10]. However, the effects of CS algorithms on image quality, spatial and spectral resolution, and level of distortion of the spectral peaks in HiSS MRI are not known. The effects on spectral resonance lineshape are especially important since the spectral analysis of HiSS MRI has produced strong diagnostic performance in prior work, with receiver operating characteristic area under the curve (ROC AUC) values of up to 0.90 [7,8,9]. Characterizing the performance of SENSE and CS algorithms could guide the future selection of the acceleration method optimized for coil geometry and/or intended data analysis.

This manuscript aims to characterize the effect of CS on noise and artifact levels, spatial and spectral resolution, and spectral resonance lineshape in HiSS MRI and compare it to that of SENSE acceleration, with increasing acceleration factors of up to R = 10.

## 2. Methods

### 2.1. Imaging Phantom

**Figure 1:**
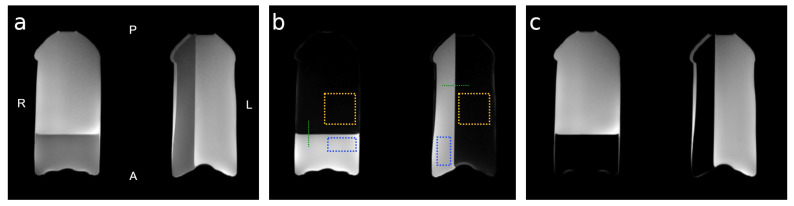
The physical layout of the agar/oil phantom and regions of interest (ROIs) used in analysis. In-phase (**a**), water (agar (**b**)), and fat (oil (**c**)) reference images of the agar-oil phantom obtained using a T1-weighted mDixon sequence are shown. The anterior/posterior (A/P) and left/right (L/R) orientation of the image is labeled in (**a**). The phantom was designed to provide agar gel boundaries orthogonal to the readout (anterior/posterior) and phase-encoding (left/right) directions. The agar gel is depicted in the water image (**b**). The dashed lines (green) indicate the position of the edge profiles used for MTF analysis. The dashed rectangles (blue) outlines indicate the location of the ROIs used in the noise level and spectral characteristic analysis. The dashed squares (yellow) outlines indicate the location of the ROIs used in the artifact level analysis.

Imaging phantoms were prepared from degassed 2% agarose solution in nano-filtered distilled water. The solution was degassed for 30 min to remove air bubbles which could create inhomogeneities that are detrimental to T2* imaging inherent in HiSS MRI. Two bottles were partially filled and cooled, one in a vertical and one in a horizontal position, to provide two different phantom geometries. The remaining volume was filled with vegetable oil to minimize the susceptibility gradient across the surface of the agar-filled portion of the phantom. One bottle was placed in each of the volumes of a dedicated 15-channel breast coil and secured to minimize vibration during imaging. The phantom was positioned such that the agar-oil boundary was perpendicular to the readout (anterior/posterior) direction in the right (image left) and the phase encoding (left/right) direction in the left (image right) breast volume. Figure 1 depicts the phantom in the axial cross-section, shows the agar and oil compartments separately, and outlines the locations of the edge profiles and regions of interest (ROIs) used in the analysis, as described below.

### 2.2. Data Acquisition

Imaging was conducted on a 3T dStream Ingenia scanner (Philips, Amsterdam, The Netherlands), using a dedicated, closed, dStream 16-channel breast coil (Philips, Amsterdam, The Netherlands). The dStream digital architecture is equipped with a high number of independent channels with direct digitization at the coil elements. Axial mDixon T1-weighted images were acquired for reference. Axial images through the center of the phantom were acquired using the High Spectral and Spatial (HiSS) MRI sequence, which was based on a 2D echo-planar spectroscopic imaging (EPSI) sequence [34,35] (FOV 256 × 384 × 3 mm^3^; spatial resolution 0.8 × 0.8 × 3 mm^3^; TR/TE/ΔTE 1000/122/1.89 ms, flip angle 90°; echo train length 127; spectral resolution 4.17 Hz, IB-autoshim), with SENSE acceleration factors of 1, 2, 3, 4, 6, 8, and 10. The HiSS sequence was implemented via a software patch. Four slices central to the phantom were acquired, and only one central slice was analyzed. At the SENSE acceleration factor of 1, HiSS MRI was acquired with full k-space coverage on a Cartesian grid. The complex raw k-space data for each coil element were exported individually for CS post-processing. All acquisitions were repeated five times to allow for the evaluation of variability and noise levels.

### 2.3. SENSE and CS Reconstruction

SENSE reconstruction was performed on the console with the scanner-provided dS SENSE algorithm to obtain gradient echo images at 127 individual TEs. dS SENSE utilizes the dStream architecture by including a smart selection of coil elements [36]. Dual-calibration reference scan, minimal artifact factor algorithm [37], and anatomy-based regularization [38]. Complex MR images (real and imaginary components) were exported for post-processing. CS reconstruction was performed offline, as follows. k-space variable-density random undersampling of HiSS MRI data was simulated using Cartesian k-space undersampling masks that eliminated whole k-space lines along the readout direction and were constant for all echoes. These masks differed from SENSE undersampling masks, as the latter are typically regular. In contrast, CS undersampling is random by design. Full k-space information and complex gradient echo images at 127 individual TEs were then reconstructed for acceleration factors R of 2, 3, 4, 6, 8, and 10 using a distributed multi-sensor implementation [39] of CS [40] including sparsifying operators in space (Daubechies wavelet of 2nd order and total variation). Compressed sensing iterative reconstruction was implemented by means of a nonlinear conjugate gradient descent algorithm with a backtracking line search [40]. Reconstruction of multi-coil MR imagery was performed by an adaptive implementation of the spatial matched filter [41]. Complex MR images (real and imaginary components) were then exported for post-processing. As coil sensitivity maps are acquired in a separate sequence, the nominal acceleration factors describing the k-space undersampling also represent the true reduction in imaging times.

### 2.4. HiSS MRI Post-Processing

For each set of 127-echo gradient echo images reconstructed at R = 1, 2, 3, 4, 6, 8, and 10, a Fourier transform in the temporal direction was performed in each voxel to obtain the water and oil resonance spectra. Nyquist ghosting was corrected by applying a voxel-by-voxel optimized zero-order (constant) phase correction to each odd echo only. WPH images were constructed, in which the fat signal was suppressed, resulting in a sharp signal edge orthogonal to either the readout or the phase encoding direction (Figure 1).

### 2.5. Image Quality Evaluation

For image quality evaluation, WPH images obtained in HiSS post-processing, rather than gradient echo images obtained directly from acceleration algorithms, were considered. To evaluate noise levels, two 51 × 26 voxel ROIs placed in the agar area were considered (Figure 1b, blue). The size of the ROIs was selected so that they could be positioned approximately symmetrically in the two coil volumes. To remove spatial gradients and allow pure noise quantification, images were normalized to the average of acquisitions 1–5. Further, to correct for small differences (~1–3%) in hardware scaling between acquisitions 1–5, the ROI average of the normalized values was subtracted for each acquisition separately. The noise levels were then quantified as $1/\;\sqrt{2}$ of the standard deviation of the normalized and corrected image intensity over acquisitions 1–5, averaged over the agar ROIs.

To evaluate the geometric artifact level, two 51 × 51 voxel ROIs placed in the area of suppressed oil signal were considered (Figure 1b, yellow). The ROIs’ size and location were selected so that they were positioned symmetrically in the two coil volumes. The artifact levels were calculated as the mean signal intensity, averaged over acquisitions 1–5. To account for different scaling of SENSE- and CS-reconstructed HiSS images, the artifact levels were normalized to the level measured at R = 1.

### 2.6. Spatial Resolution Evaluation

**Figure 2:**
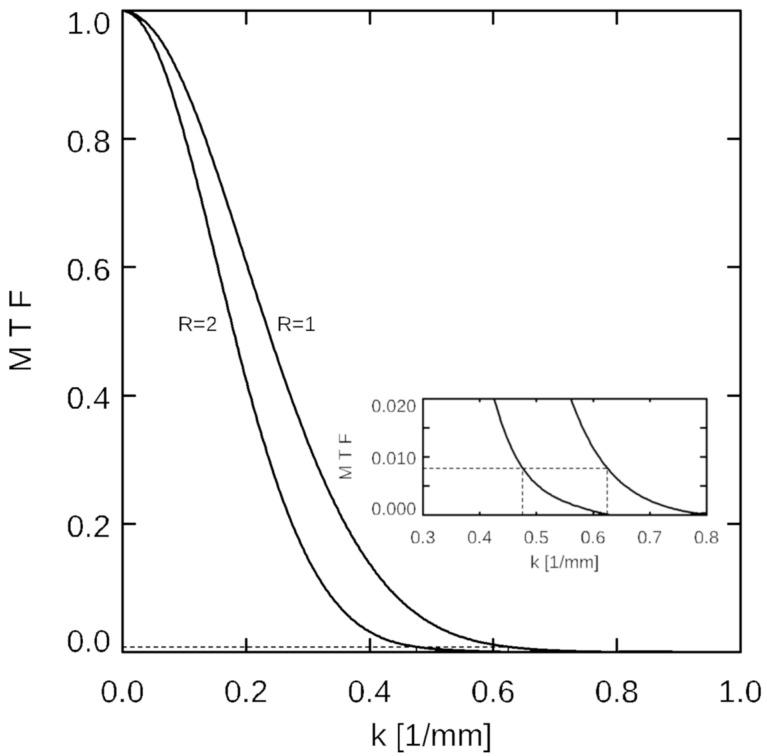
Illustration of the modulation transfer function (MTF) analysis. MTFs in the phase encoding direction are shown against wavenumber k for acceleration factors R = 1 and R = 2, for compressed sensing- (CS-) reconstructed HiSS MRI data. At k_n_ = 0.625 1/mm (corresponding to the nominal in-plane resolution of 0.8 mm), the corresponding MTF value at R = 1 is 0.0081. For R = 2, this MTF value corresponds to k = 0.48 1/mm (see inset) or an equivalent resolution of 1.05 mm.

The effect of the acceleration algorithms on effective image resolution was quantified via analysis of the modulation transfer function (MTF) [42,43]. HiSS MRI WPH signal profiles across the lateral (orthogonal to phase encoding direction) and anterior (orthogonal to readout direction) agar-oil boundary of the phantom were extracted at representative locations, illustrated in Figure 1b (green), to model a sharp edge. The spatial derivatives of the edge signal profiles were fit to a Gaussian function whose Fourier transform provided the MTF. The nominal wavenumber k_n_ was calculated from the nominal image resolution of 0.8 mm in-plane using the relation in Equation (1):

(1) $$k=\frac{1}{2\cdot \mathrm{resolution}}$$

and the value of MTF_n_ = MTF(k_n_) is recorded. Equivalent image resolution was calculated using Equation (1) for each acceleration factor R from the value of k_e_ at which the MTF(k_e_) = MTF_n_, as illustrated in Figure 2.

### 2.7. Spectral Lineshape Analysis

To analyze the dependence of the spectral characteristics of the water resonance on the acceleration factor R, two 51 × 26 voxel ROIs placed in the agar area were considered (Figure 1b, blue). The water resonance in each voxel was fit to a complex Lorentzian function:

(2) $$L\left(f\right)=\frac{A\cdot \mathrm{FWHM}}{\mathrm{FWHM}+2i\left(f-f_{0}\right)}\cdot e^{i\varphi }+{\mathrm{Be}}^{i\Phi \left(f-f_{0}\right)}$$

where $f_{0}$ is the central frequency of the resonance, FWHM is the full width at half maximum of the resonance, and amplitude A is set such that the function is normalized to $L\left(f_{0}\right)=1$. The factor $e^{i\varphi }$ accounts for phasing of the spectral resonance, and ${\mathrm{Be}}^{i\Phi \left(f-f_{0}\right)}$ accounts for the baseline with a possible linear gradient Φ in phase. FWHM was recorded as the measure of peak broadening.

**Figure 3:**
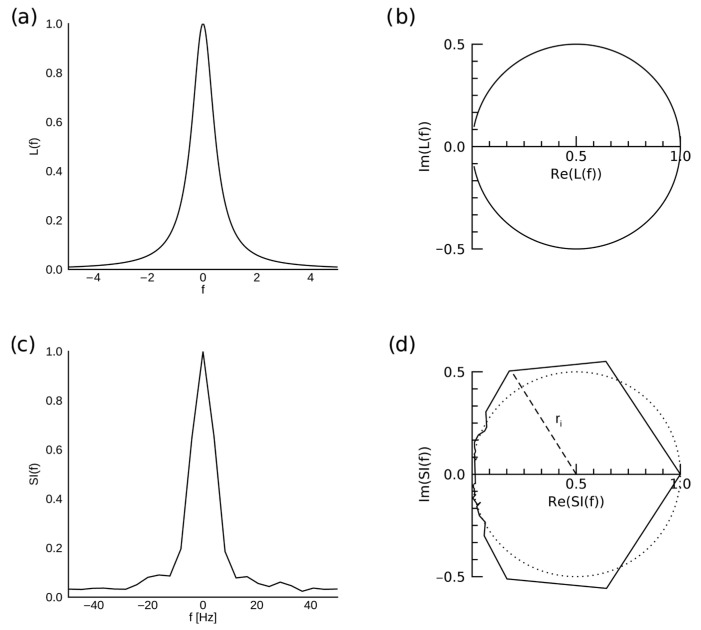
Illustration of DISPA analysis. A spectral peak with an ideal Lorentzian shape L(f), normalized so that the maximum value is 1 (**a**) is represented by a circle of radius 0.5 centered on (0.5, 0) in the complex plane (**b**). Spectral intensity SI(f) of actual water resonance from a voxel in the agar compartment of the phantom, reconstructed with CS at R = 10, is shown in (**c**), and its representation in the complex plane is shown in (**d**), with the ideal Lorentzian shape represented by the dotted circle. A radius r_i_ corresponding to one of the spectral bins is shown by the dashed line.

Further, the dispersion vs. absorption (DISPA) analysis was used to characterize the water resonance structure as a degree of departure from the ideal Lorentzian shape, quantified as Total Radial Difference (TRD) [9,44,45]. When the dispersion (imaginary) component of a phased spectral peak with ideal Lorentzian shape ($e^{i\varphi }=1$, ${\mathrm{Be}}^{i\Phi \left(f-f_{0}\right)}=0$) normalized to peak amplitude of 1 is plotted against its absorption (real) component, a perfect circle of radius 0.5 centered at (0.5, 0) is obtained (Figure 3). Then, TRD is calculated as:

(3) $$\mathrm{TRD}=\underset{b}{\sum }\left(r_{b}-0.5\right)$$

where b indexes each spectral bin in the 57.5 Hz (0.45 ppm) neighborhood of the spectral resonance, and $r_{b}$ is the radial distance from the center of the DISPA circle. Equivalently:

(4) $$\mathrm{TRD}=\underset{b}{\sum }\left|L\left(f-f_{0}\right)-\frac{1}{2}\right|-\frac{1}{2}$$

The phasing of the resonance was performed, and the baseline was removed in each voxel. TRD was calculated and averaged over the two ROIs.

Because TRD is correlated with kurtosis of the spectral peak and not sensitive to asymmetry or the presence of multiple peaks, Total Absolute Radial Difference (TARD) was also calculated as:

(5) $$\mathrm{TARD}=\underset{b}{\sum }|\left|L\left(f-f_{0}\right)-\frac{1}{2}\right|-\frac{1}{2}|$$

and averaged over the two ROIs. Since in TARD, all deviation from the Lorentzian shape is compounded by adding absolute values, rather than averaged by adding signed (positive or negative) values, TARD is more sensitive than TRD to the presence of off-peak components, asymmetry, and skewness.

## 3. Results

**Figure 4:**
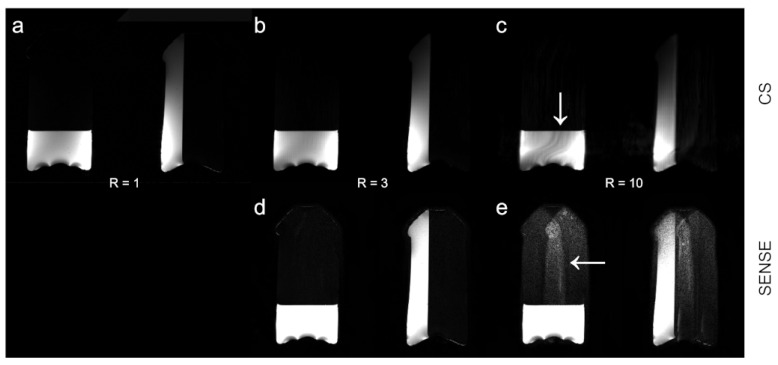
Illustration of artifacts apparent at high R levels. HiSS MRI water peak height (WPH) images are shown for increasing acceleration factors R for CS and SENSE acceleration methods. WPH images for (**a**) R = 1, (**b**) CS R = 3, (**c**) CS R = 10, (**d**) SENSE R = 3, and (**e**) SENSE R = 10 are depicted. The WPH images are scaled to illustrate slight spatial inhomogeneity in the image-left volume of the phantom in CS images and the amplification of the geometric artifact in SENSE-accelerated images at R = 10. The artifacts are indicated by arrows.

Figure 4 shows HiSS WPH images at R = 1 and reconstructed at R = 3 and R = 10, using the CS (top) and SENSE (bottom) algorithms. At R = 10, increased signal intensity modulation is visible in the CS-reconstructed image, while geometry-related reconstruction artifacts are visible in the SENSE-accelerated image.

### 3.1. Image Quality Evaluation

**Figure 5:**
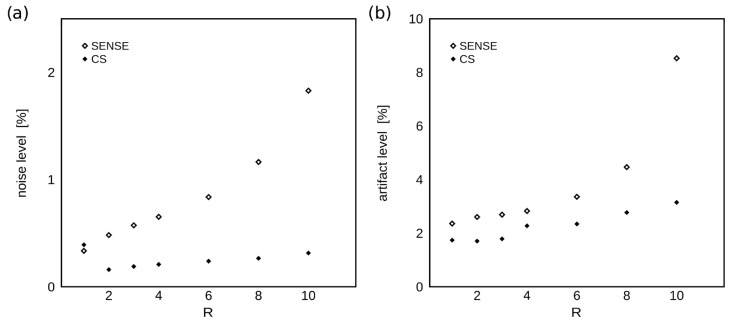
Water peak height image quality measures as a function of R. (**a**) The dependence of the noise level (relative to agar signal) on the acceleration factor R is shown for SENSE and CS acceleration schemes. (**b**) The dependence of the geometric artifact level (relative to agar signal) on R is shown for SENSE and CS acceleration schemes. The noise and artifact levels are relatively constant or increase minimally with R for CS but increase approximately 5.5-fold and 4-fold, respectively, for SENSE-accelerated imaging at R = 10.

Figure 5 shows the noise levels and relative artifact levels of WPH images at increasing acceleration factors, R for SENSE and CS acceleration algorithms. The noise level was suppressed and approximately constant with R for CS-accelerated imaging, while SENSE acceleration significantly amplified noise, approximately 5.5-fold for R = 10 (Figure 5a). Similarly, the geometric artifact level rose minimally with R under CS acceleration but increased above 8%, or approximately 4-fold, for SENSE acceleration factor of 10 (Figure 5b).

### 3.2. Spatial Resolution Evaluation

**Figure 6:**
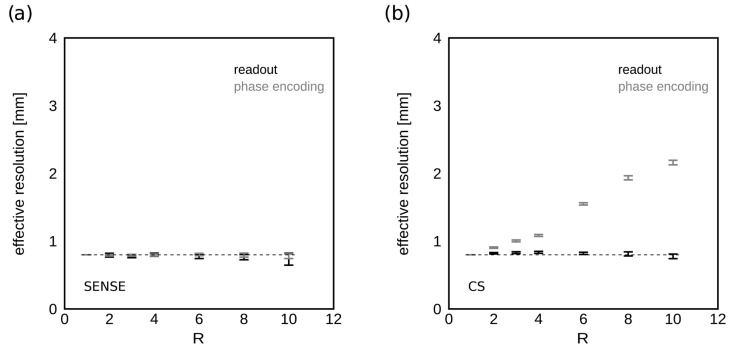
Spatial resolution as a function of R. The dependence of effective spatial resolution in the (**a**) SENSE- and (**b**) CS-accelerated HiSS MRI WPH images on the acceleration factor R is shown for the readout and phase encoding directions. The dashed lines indicate the nominal spatial resolution of 0.8 mm in each direction. SENSE acceleration preserves spatial resolution. For CS, the loss of resolution in the phase encoding direction is noticeable, up to a factor of 2.7 for 10-fold acceleration.

Figure 6 shows the calculated effective resolution derived from the MTF analysis at increasing acceleration factors R for SENSE (Figure 6a) and CS (Figure 6b) acceleration algorithms. SENSE acceleration did not degrade the effective spatial resolution of HiSS MRI, which was nominally 0.8 in-plane and measured as 0.78 ± 0.02 mm in the readout direction, and 0.79 ± 0.01 mm in the phase encoding direction. Under CS reconstructions, the effective spatial resolution was 0.81 ± 0.02 mm in the readout direction, and 0.80 mm, 0.91 mm, 1.00 mm, 1.08 mm, 1.55 mm, 1.94 mm, and 2.16 mm in the phase encoding direction, for R = 1, 2, 3, 4, 6, 8, and 10, respectively.

### 3.3. Spectral Resolution Evaluation

**Figure 7:**
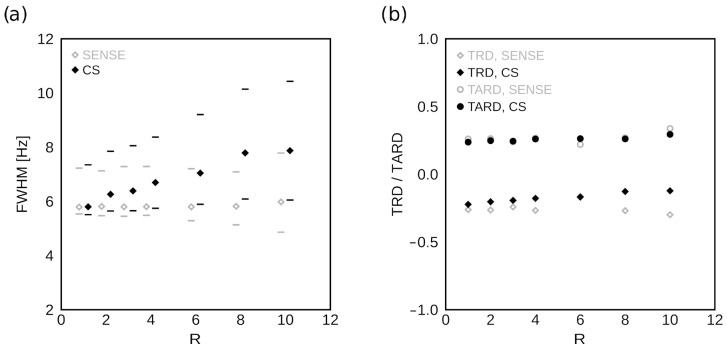
Spectral lineshape descriptors as a function of R. (**a**) The dependence of the ROI-averaged FWHM on the acceleration factor R is shown for SENSE- (gray) and CS-based (black) acceleration. The horizontal lines represent the minimum and maximum values of FWHM in the ROI used for analysis. There is little variation with R in the minimal FWHM values or under SENSE acceleration generally. (**b**) The dependence of TRD and TARD on the acceleration factor R is shown for SENSE- (gray) and CS-based (black) acceleration. While the effects of increasing R on TRD diverge between SENSE and CS, they are minimal. TARD shows virtually no variation with R for either acceleration method.

Figure 7a shows the dependence of the ROI-averaged FWHM on the acceleration factor R for SENSE and CS. The minimum and maximum FWHM in the ROI is also noted. There is minimal variation in the minimum values of FWHM, or generally in SENSE-accelerated data. In CS-accelerated data, the average and the maximum values of FWHM increase by up to approximately 35% and 40%, respectively, at R = 10. Figure 7b shows the dependence of TRD and TARD on the acceleration factor R. TRD shows little difference between SENSE- and CS-accelerated data and minimal variation with R. In contrast, TARD shows virtually no dependence on R for either acceleration method.

Table 1 summarizes the differences between the SENSE and CS acceleration methods and their expected advantages/disadvantages on clinical diagnosis.

## 4. Discussion

We have examined the effects of SENSE- and CS-based acceleration schemes on image quality and spectral characteristics of the HiSS MRI data using an agar-oil phantom. Artifact levels track similarly between SENSE and CS, up to about R = 6, but SENSE clearly amplifies the noise levels, while under CS, they are suppressed, which can yield higher image quality. SENSE-generated artifacts are geometry-related, with often sharp edges between areas of high and low artifact levels at high R, as illustrated in Figure 4, which can affect the morphological analysis. These artifacts arise from the geometric factor g, which is derived from coil sensitivity maps and used in SENSE reconstruction. Under CS, some image intensity modulation can be observed, as illustrated in Figure 4, but its low spatial frequency would not affect the local evaluation of morphology. This modulation is likely arising from line broadening in areas of high B0 inhomogeneity. Similarly, as spectral analysis is not scale-dependent, it would not be affected by the CS-related intensity modulation artifact.

Conversely, SENSE does an excellent job of preserving spatial resolution in both directions, while under CS, the spatial resolution in the phase-encoding direction is decreased starting at R = 3 and clearly compromised at R = 6. Most importantly, SENSE can be expected to do an excellent job of preserving the spectral resonance lineshape, as evidenced by FWHM, TRD, and TARD being approximately constant with R, while CS increases the FWHM with increasing R. This is driven by the increase in the maximum FWHM values, as the minimum FWHM is not changed by R. This is a likely result of the reduced spatial resolution, which leads to mixing of the signal in neighboring voxels; when B0 gradients are present, this can lead to peak broadening. It is not implausible that higher acceleration factors would have been practical with CS than with SENSE, which led us to examine the cases of R > 4, typically not used in clinical applications. This would have been the case if the spatial resolution penalty was lower than what we observed.

There are at least two distinct reasons for observing a lower effective resolution in CS-accelerated images. One, the effect could be directly related to blurring due to reconstructing k-space data with missing phase-encoding lines. In addition, the WPH image intensity is a function of peak broadening—a peak with higher FWHM will have a lower peak height and vice versa, which could result in a reduction in observed effective resolution at boundaries where there is a magnetic susceptibility gradient present. However, such peak broadening would affect boundaries in both directions (perpendicular to readout and phase encoding) equally, which is not observed. Thus, the reduction in effective spatial resolution is ascribed to blurring due to undersampling in the phase encoding direction.

In this work, the reduced spatial resolution did not have a notable effect on the spectral lineshape characterized by TRD and TARD. However, this is not unexpected in a uniform phantom without distinct sub-voxel water compartments and spatial inhomogeneities. In a biological sample, where voxel-to-voxel variation is not only possible but is the source of the diagnostic information, signal mixing between neighboring voxels could be detrimental to the diagnostic performance. Spectral broadening and distortion can affect the asymmetry [4,5,10], off-peak component [6,7], and DISPA analyses [9], which have been used to produce biomarkers of disease. In addition, when imaging a human subject, the shimming may be less effective, and stronger B0 gradients may be present, causing more pronounced spectral blurring. Thus, the observed effects on water resonance lineshape represent lower estimates.

Based on the results of this study, with acceleration factors of up to R = 4, SENSE and CS perform similarly. However, in biological samples, the noise amplification under SENSE acceleration is likely to be more consequential due to lower SNR levels. Conversely, degradation of water resonance structure under CS will likely be more readily observable.

There are several limitations to this study. One, the on-scanner implementation of CS for HiSS MRI is currently not available. Thus, CS acceleration was simulated, and all R > 1 image sets were produced from the same complete k-space dataset. SENSE-accelerated image sets were acquired as separate sequences, potentially introducing additional variation. Second, the measures of spectral structure used here are sensitive to changes in peak width, kurtosis, asymmetry, and skewness overall. Still, evaluation of fine structure of the water resonance was not possible with this phantom. This could be addressed in the future by using a fixed tissue sample. Finally, the SNR levels in phantom studies are typically much higher than what is achievable in clinical MRI exams. Thus, the trade-off between SENSE and CS acceleration algorithms should be evaluated separately in a clinical context.

## 5. Conclusions

In conclusion, we have characterized the behavior of noise and image artifact levels, spatial and spectral resolution, and lineshape characteristics with increasing acceleration factors up to R = 10 for SENSE and CS acceleration methods. CS has shown better performance in noise and artifact suppression. At the same time, the fidelity of spectral lineshape characteristics could be compromised due to a reduced spatial resolution in the phase encoding direction. Thus, CS might show advantages in applications that are noise-limited or geometrically constrained, such as prostate imaging without an endo-rectal coil. In geometric configurations specific to breast MRI, SENSE-based acceleration may allow for better diagnostic performance when HiSS MRI is used.

## Figures and Tables

**Table 1:** Comparison of SENSE vs. CS properties under increasing acceleration factors.

Property	SENSE	CS	Likely Effect on Clinical Diagnosis
Artifact type	Noise amplification	Intensity modulation	CS preferred due to the spatially gradual artifact nature
Noise level	Increased	Suppressed	CS would allow more robust analysis
Artifact level in water-suppressed region	Higher	Lower	CS would allow more robust analysis
Spatial resolution	Preserved	Degraded in the phase-encoding direction	CS might compromise morphological/radiomics analysis
Spectral line width	Preserved	Degraded in areas of high B0 inhomogeneity	CS might compromise spectral analysis
Spectral line shape	Preserved	Preserved	Neither method is preferred

CS—compressed sensing; SENSE—sensitivity encoding.

## Data Availability

The data presented in this study are available on request from the corresponding author. The data are not publicly available due to the size and complexity of the dataset.
